# Synergistic Anti-Obesity Effects of *Lactiplantibacillus plantarum* Q180 and *Phaeodactylum tricornutum* (CKDB-322) in High-Fat-Diet-Induced Obese Mice

**DOI:** 10.3390/ijms26167991

**Published:** 2025-08-19

**Authors:** Hye-Ji Noh, Jae-In Eom, Soo-Je Park, Chang Hun Shin, Se-Min Kim, Cheol-Ho Pan, Jae Kwon Lee

**Affiliations:** 1 Probiotics Research Laboratory, Chong Kun Dang Bio (CKDBio) Research Institute, Ansan 15604, Republic of Korea; nhj@ckdbio.com (H.-J.N.); sjepark@ckdbio.com (S.-J.P.); chshin@ckdbio.com (C.H.S.); 2 Microalgae Ask Us Co., Ltd., Gangneung 25441, Republic of Korea; umjaein@maus2020.com (J.-I.E.); kimsemin@maus2020.com (S.-M.K.); 3 Department of Biology Education, College of Education, Chungbuk National University, Cheongju 28644, Republic of Korea

**Keywords:** probiotics, microalgae, anti-obesity, *L. plantarum*, *P. tricornutum*

## Abstract

Obesity and associated metabolic disorders are rising globally, necessitating effective dietary strategies. CKDB-322, a formulation containing *Lactiplantibacillus plantarum* Q180 and *Phaeodactylum tricornutum*, was evaluated for anti-obesity efficacy using in vitro adipocyte differentiation and in vivo high-fat-diet (HFD)-induced obese mouse models. In 3T3-L1 cells, CKDB-322 suppressed adipogenesis by downregulating PPARγ and C/EBPα and enhancing glycerol release. In mice, 8 weeks of oral administration—particularly at the CKDB-322-M dose—significantly reduced body weight gain, adiposity, and serum glucose, triglyceride, and cholesterol levels without affecting liver function. Gene expression analysis revealed the strong inhibition of lipogenic markers (SREBP-1c, ACC, and FAS) in addition to activation of the fatty acid oxidation (CPT-1α and PPARα) and energy metabolism (PGC-1α and AMPK) pathways, with the most pronounced effects in the CKDB-322-M group, which also exhibited the greatest reduction in leptin. These molecular effects were confirmed histologically by decreased adipocyte hypertrophy and ameliorated hepatic steatosis. Collectively, these findings demonstrate that CKDB-322 exerts lipid-modulatory effects through multiple pathways, supporting its potential as a novel functional dietary ingredient for obesity and metabolic disorder prevention.

## 1. Introduction

Obesity is a pathological condition characterized by the excessive accumulation of body fat [1]. It has become a global epidemic, affecting more than 650 million adults worldwide and contributing to the growing burden of metabolic disorders, including type 2 diabetes, metabolic dysfunction-associated steatotic liver disease (MASLD), and cardiovascular complications [2]. Excessive energy intake leads to the accumulation of triglycerides within adipocytes, resulting in adipocyte hypertrophy and hyperplasia. As this expansion of adipose tissue persists, it progressively contributes to the development of obesity. This pathological process is closely associated with the inactivation of AMP-activated protein kinase (AMPK), a key metabolic regulator whose suppression promotes adipogenesis and lipogenesis while inhibiting fatty acid β-oxidation [3,4,5]. Due to the adverse effects and limited long-term efficacy of conventional pharmacological treatments, increasing attention has been directed toward food-derived bioactive compounds as potentially safer and more sustainable alternatives for obesity management [6].

Probiotics are defined as live microorganisms that, when administered in adequate amounts, confer health benefits to the host [7]. Given that the composition of the gut microbiota plays a pivotal role in host metabolism, the probiotic-induced modulation of the microbial community may contribute to broader metabolic improvements beyond their originally observed effects. Previous studies have shown that probiotics can improve metabolism through several molecular mechanisms, including modulating gut microbiota composition, enhancing short-chain fatty acid production, regulating gut hormones, and influencing host immune responses. These actions can lead to improved insulin sensitivity, better lipid metabolism, and reduced inflammation, all of which are crucial for metabolic health [8]. Emerging evidence suggests that probiotics may enhance host metabolism via gut–organ axes such as the gut–brain axis, partly by improving leptin sensitivity through microbiota-driven anti-inflammatory and hormonal effects [9]. *Lactiplantibacillus plantarum* (*L. plantarum*), a bacterium, which is a widely distributed species within the genus *Lactiplantibacillus* that is commonly found in fermented food products and anaerobic plant materials [10,11], has demonstrated beneficial effects on body weight, insulin resistance, and lipid profiles, primarily through the modulation of the gut microbiota [12,13,14]. In addition, *L. plantarum* can influence both innate and adaptive immune responses, acting as an immune adjuvant. It can regulate cytokine production, influencing the balance of immune cells like T helper cells [15]. Although the effect of *L. plantarum* Q180, a strain isolated from the feces of healthy adults, on postprandial triglyceride (TG) levels has been clearly demonstrated [16,17], its potential to improve other lipid-related parameters, such as body fat accumulation, remains of interest and warrants further investigation.

Marine microalgae have attracted considerable attention due to their rich nutritional and functional properties [18]. Cultured microalgae are known to produce more than 15,000 bioactive compounds, including fatty acids, sterols, phenolic compounds, terpenes, enzymes, polysaccharides, alkaloids, toxins, and various pigments such as lutein and β-carotene. *Phaeodactylum tricornutum* (*P. tricornutum*), a diatom containing eicosapentaenoic acid (EPA) and fucoxanthin as the major bioactive compounds, and polyphenols as supplementary bioactive components [19], has demonstrated anti-obesity effects in both in vitro and in vivo studies. These effects include the suppression of adipocyte differentiation, the modulation of hepatic lipid metabolism, and reductions in oxidative stress [20]. In addition, *P. tricornutum* has been reported to exert anti-inflammatory, antioxidant, neuroprotective, and gut microbiota-modulating effects [21,22,23,24,25]. Microalgae-derived EPA offers several advantages over fish oil, including sustainability, reduced risk of contaminants, and suitability for vegan diets. Algal oil is a renewable source of EPA, mitigating the environmental impact of overfishing and supporting marine ecosystems. It also avoids potential contaminants like mercury and PCBs that can be found in some fish oils [26].

Although *L. plantarum* Q180 and *P. tricornutum* have each demonstrated anti-obesity potential, their combined effects remain largely unexplored. *L. plantarum* Q180 has been shown to reduce body fat by modulating gut microbial composition, whereas *P. tricornutum* contains bioactive compounds that directly influence lipid metabolism. Given these distinct yet complementary mechanisms, their co-administration may produce synergistic effects on the management of adiposity. Therefore, this study aimed to evaluate the anti-obesity efficacy of combined *L. plantarum* Q180 and *P. tricornutum* treatment in a high-fat-diet-induced obese mouse model.

## 2. Results

### 2.1. Characterization of P. tricornutum Using Gas Chromatography–Flame Ionization Detection (GC-FID)

To ensure quality control, the fatty acid composition of *P. tricornutum* was analyzed by GC-FID. The quantitative analysis demonstrated that *P. tricornutum* contained 3194.23 ± 81.27 mg/100 g of EPA, confirming the consistent and measurable presence of this bioactive compound (Table 1).

### 2.2. Effect of CKDB-322 on the mRNA Expression of Adipogenic Transcription Factors in 3T3-L1 Adipocytes

**Figure 1:**
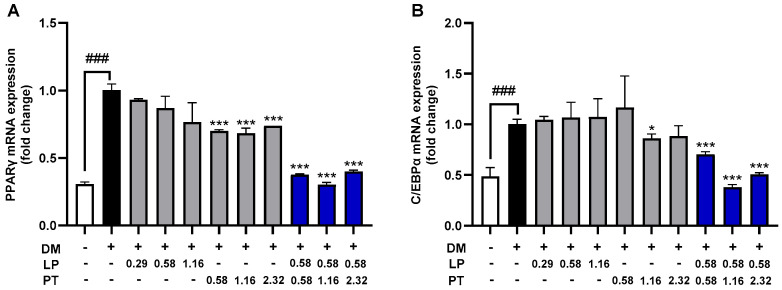
mRNA expression of adipogenic transcription factors PPARγ (**A**) and C/EBPα (**B**). Gene expression levels are measured using reverse transcription-quantitative polymerase chain reaction (RT-qPCR) and normalized to β-actin. Data are expressed as the means ± standard deviations (SD), *n* = 3 per group. ^###^ *p* < 0.001 vs. undifferentiated control (pre-adipocytes); * *p* < 0.05, *** *p* < 0.001 vs. DM-only group (differentiated adipocytes). LP was treated at 0.29, 0.58, and 1.16 mg/mL, corresponding to 1 × 10^8^, 2 × 10^8^, and 4 × 10^8^ CFU/mL of heat-killed *L. plantarum* Q180, respectively, using both the pellet and its culture supernatant. PT was treated at 0.58, 1.16, and 2.32 mg/mL of lyophilized *P. tricornutum*. The mixture of LP and PT (CKDB-322) was applied at the indicated concentrations (mg/mL). Please refer to Supplementary Table S1 for the raw data.

The effect of CKDB-322 on adipogenesis was evaluated by measuring the mRNA expression levels of two key adipogenic transcription factors, peroxisome proliferator-activated receptor gamma (PPARγ) and CCAAT/enhancer-binding protein alpha (C/EBPα), in 3T3-L1 adipocytes using reverse transcription-quantitative polymerase chain reaction (RT-qPCR). As shown in Figure 1, the expression levels of PPARγ and C/EBPα were significantly upregulated in the differentiation medium (DM)-only group (differentiated adipocytes) compared to undifferentiated controls (pre-adipocytes), by approximately 3.27-fold and 2.06-fold, respectively (*p* < 0.001). Treatment with CKDB-322 at all tested concentrations significantly suppressed the expression of both genes compared to the DM-only group (*p* < 0.001). In contrast, treatment with *L. plantarum* Q180 (LP) or *P. tricornutum* (PT) alone showed only partial or minimal inhibitory effects. Importantly, no cytotoxicity was observed at any of the tested concentrations of LP, PT, or CKDB-322. These findings suggest that CKDB-322 potently inhibits adipogenesis by downregulating the transcriptional activation of PPARγ and C/EBPα during adipocyte differentiation.

### 2.3. Effects of CKDB-322 on Lipolytic Activity In Vitro

**Figure 2:**
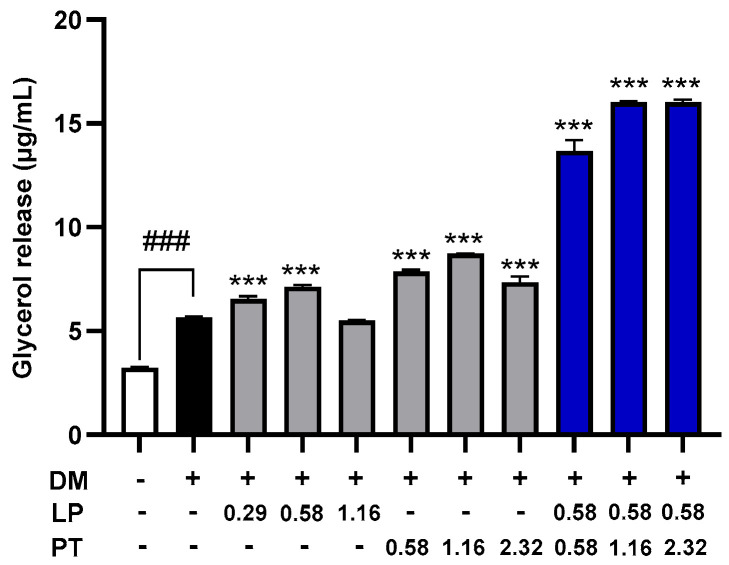
Glycerol release as a marker of lipolytic activity in 3T3-L1 adipocytes. Glycerol levels in the culture medium were measured in differentiated 3T3-L1 adipocytes following treatment with LP, PT, or their mixture (CKDB-322) at the indicated concentrations (mg/mL). LP was treated at 0.29, 0.58, and 1.16 mg/mL, corresponding to 1 × 10^8^, 2 × 10^8^, and 4 × 10^8^ CFU/mL of heat-killed *L. plantarum* Q180, respectively, using both the pellet and its culture supernatant. PT was treated at 0.58, 1.16, and 2.32 mg/mL of lyophilized *P. tricornutum*. The mixture of LP and PT (CKDB-322) was applied at the indicated concentrations (mg/mL). Data are expressed as the means ± standard deviations (SD), *n* = 3 per group. ^###^ *p* < 0.001 vs. undifferentiated control (pre-adipocytes); *** *p* < 0.001 vs. DM-only group (differentiated adipocytes).

Lipolytic activity was evaluated by measuring glycerol release into the culture medium as a marker of triglyceride (TG) hydrolysis in differentiated 3T3-L1 adipocytes. CKDB-322 at 1.74 mg/mL induced the highest level of glycerol release (~16 μg/mL), which was significantly higher than that observed in the DM-only group and in cells treated with LP or PT alone at equivalent concentrations (*p* < 0.001; Figure 2). These results suggest that the combination of LP and PT in CKDB-322 exerts a synergistic effect on enhancing lipolysis in adipocytes.

### 2.4. Effect of CKDB-322 on Body Weight Gain, Food Intake, and Feed Efficiency Ratio (FER) in High-Fat-Diet (HFD)-Induced Obese Mice

To evaluate the effects of CKDB-322 on obesity in vivo, an obese animal model was created and used in the experiment. After the 8-week experimental period, mice in the HFD group showed a significantly greater increase in body weight (16.4 ± 3.2 g) compared to the normal-diet (ND) group (7.1 ± 1.9 g; *p* < 0.001), confirming the obesogenic effect of the HFD. CKDB-322 supplementation significantly attenuated body weight gain at all tested doses (L, M, and H) relative to the HFD group. Among them, the CKDB-322-H group (10.7 ± 2.2 g) showed the most pronounced reduction (34.8%), exceeding the effects of LP (11.8 ± 1.7 g) and PT (13.2 ± 2.2 g) and suggesting a synergistic or additive anti-obesity effect at a high dose.

Food intake was significantly reduced in the LP, PT, and all CKDB-322-treated groups compared to the HFD group (*p* < 0.001), while no significant change was observed in the *Garcinia cambogia* extract (GC) group. Despite similar food intake levels, the FER was significantly lower in all CKDB-322 groups. Notably, the CKDB-322-H group exhibited the lowest FER (0.092 ± 0.0) compared to the HFD group (0.133 ± 0.0; *p* < 0.001), indicating improved metabolic efficiency (Table 2). Please refer to Supplementary Table S2 for the raw data.

### 2.5. Effects of CKDB-322 on Serum Biochemical Parameters in HFD-Induced Obese Mice

Blood glucose levels were significantly elevated in the HFD group (221.9 ± 23.9 mg/dL) compared to the ND group (168.5 ± 36.7 mg/dL; *p* < 0.01), confirming hyperglycemia associated with HFD-induced obesity. This elevation was significantly attenuated in the GC, PT, CKDB-322-L, and CKDB-322-M groups compared to the HFD group (Table 3). Among them, the CKDB-322-M group exhibited the most pronounced glucose-lowering effect, with greater reduction than either the LP or PT single-compound groups, suggesting a potential synergistic effect of the combined formulation.

Serum triglyceride (TG) levels were significantly elevated in the HFD group (61.1 ± 6.4 mg/dL) compared to the ND group (47.1 ± 8.9 mg/dL; *p* < 0.001). This elevation was significantly reduced only in the CKDB-322-treated groups (L, M, and H), whereas the single-compound groups (LP and PT) did not show significant changes compared to the HFD group (Table 3). Notably, the CKDB-322-M group exhibited the most pronounced TG-lowering effect, with a greater reduction than either the LP or PT group, suggesting a potential synergistic interaction of the combined components.

Serum total cholesterol levels were significantly elevated in the HFD group (149.0 ± 9.0 mg/dL) compared to the ND group (106.2 ± 12.4 mg/dL; *p* < 0.001). Among the treatment groups, the CKDB-322-M group exhibited the most pronounced cholesterol-lowering effect (117.3 ± 12.5 mg/dL; *p* < 0.001 vs. HFD), whereas total cholesterol levels increased again in the CKDB-322-H group (129.1 ± 15.8 mg/dL), suggesting a reversal of effect at the highest dose. For LDL-cholesterol, only the CKDB-322-L (13.5 ± 3.3 mg/dL; *p* < 0.05) and CKDB-322-M (14.4 ± 3.8 mg/dL; *p* < 0.05) groups showed significant reductions compared to the HFD group (18.0 ± 3.8 mg/dL). For HDL-cholesterol, the elevation was significantly attenuated in the GC (106.9 ± 15.4 mg/dL; *p* < 0.001), LP (104.0 ± 14.2 mg/dL; *p* < 0.001), CKDB-322-L (116.3 ± 10.9 mg/dL; *p* < 0.01), and CKDB-322-M (115.3 ± 10.4 mg/dL; *p* < 0.001) groups compared to the HFD group (134.4 ± 10.0 mg/dL). No significant differences were observed in AST or ALT levels among the experimental groups, indicating that these parameters were not substantially affected by the treatments (Table 3). These results indicate that the treatments did not adversely affect liver function, supporting the safety of the test substances during the study period.

### 2.6. Effects of CKDB-322 on Body Fat Mass Accumulation in HFD-Induced Obese Mice

To evaluate the effects of the test substances on adiposity, body fat percentage was measured using dual-energy X-ray absorptiometry (DEXA). As shown in Table 4, the HFD group exhibited a significant increase in body fat percentage (40.5 ± 2.0%) compared to the ND group (25.2 ± 3.6%; *p* < 0.001). All treatment groups showed a significant reduction in body fat compared to the HFD group. Notably, all CKDB-322-treated groups (L, M, and H) demonstrated significant reductions in body fat mass relative to the HFD group (*p* < 0.001), indicating a consistent anti-adiposity effect of the combined formulation. The weights of various fat depots and the liver were measured to evaluate adiposity and organ changes, as shown in Table 4. The white adipose tissue weight, including epididymal, retroperitoneal, mesenteric, and inguinal fat, was significantly increased in the HFD group (4.75 ± 0.91 g) compared to the ND group (1.53 ± 0.44 g; *p* < 0.001). All CKDB-322 treatment groups (L, M, and H) showed significant reductions in total white adipose tissue weight and individual fat depots compared to the HFD group. The CKDB-322-L group demonstrated the most pronounced anti-adiposity effect, with the largest reductions across epididymal, retroperitoneal, mesenteric, and inguinal fat. While CKDB-322-M and CKDB-322-H also significantly reduced fat mass, the magnitude of the reduction was slightly less consistent, particularly in mesenteric and inguinal fat, suggesting that the low-dose formulation may be the most effective in suppressing adipose accumulation. Liver weight was significantly increased in the HFD group (1.06 ± 0.12 g) compared to the ND group (0.95 ± 0.04 g), indicating hepatic enlargement likely due to lipid accumulation. This increase was significantly attenuated by all test substance treatments, including GC, LP, PT, and CKDB-322 supplementation. Among the treatment groups, CKDB-322-L (0.80 ± 0.07 g) and CKDB-322-M (0.81 ± 0.05 g) exhibited the greatest reductions in liver weight, both showing a similar degree of effect. These findings suggest that the combined formulation at low and medium doses may be particularly effective in alleviating hepatic lipid accumulation and improving fatty liver conditions (Table 4).

### 2.7. Effects of CKDB-322 on Histopathological Morphology of Epididymal Adipose Tissue in HFD-Induced Obese Mice

**Figure 3:**
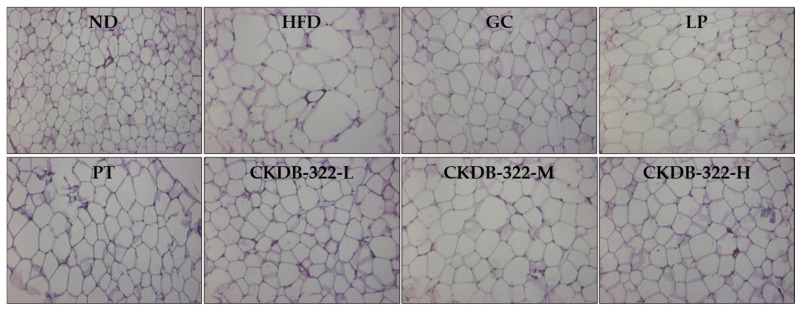
Histological analysis of epididymal adipose tissue in HFD-induced obese mice. Representative hematoxylin and eosin (H&E)-stained sections of epididymal adipose tissue (original magnification, 200×; scale bar = 100 µm) are shown for each group. ND, normal-diet group; HFD, 60% high-fat-diet group; GC, HFD supplemented with *Garcinia cambogia* extract (200 mg/kg BW); LP, HFD supplemented with *L. plantarum* Q180 (20.8 mg/kg BW equivalent to 2 × 10^8^ CFU/day); PT, HFD supplemented with *P. tricornutum* (41.7 mg/kg BW); CKDB-322-L, HFD supplemented with LP (20.8 mg/kg BW) and low-dose PT (20.8 mg/kg BW); CKDB-322-M, HFD supplemented with LP (20.8 mg/kg BW) and mid-dose PT (41.7 mg/kg BW); and CKDB-322-H, HFD supplemented with LP (20.8 mg/kg BW) and high-dose PT (83.4 mg/kg BW).

Histological analysis of epididymal adipose tissue was performed using hematoxylin and eosin (H&E) staining to evaluate adipocyte morphology following treatment (Figure 3). Adipocyte size was markedly increased in the HFD group compared to the ND group, indicating fat accumulation. In contrast, all treatment groups exhibited a reduction in adipocyte size relative to the HFD group, suggesting a mitigating effect on adipocyte hypertrophy.

**Figure 4:**
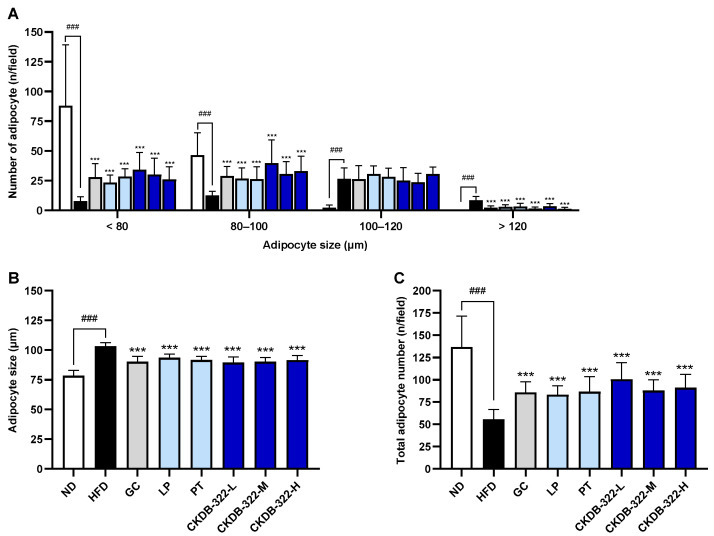
Quantitative analysis of the histopathological results of epididymal adipose tissues. (**A**) The number of adipocytes in a defined area according to their size. (**B**) Average size of adipocytes within a defined area. (**C**) Total number of adipocytes within a defined area. Values are expressed as means ± standard deviations (SD), with *n* = 10 mice per group. ^###^ *p* < 0.001 indicates significant differences compared to the ND group. *** *p* < 0.001 indicates significant differences compared to the HFD group. ND, normal-diet group; HFD, 60% high-fat-diet group; GC, HFD supplemented with *Garcinia cambogia* extract (200 mg/kg BW); LP, HFD supplemented with *L. plantarum* Q180 (20.8 mg/kg BW equivalent to 2 × 10^8^ CFU/day); PT, HFD supplemented with *P. tricornutum* (41.7 mg/kg BW); CKDB-322-L, HFD supplemented with LP (20.8 mg/kg BW) and low-dose PT (20.8 mg/kg BW); CKDB-322-M, HFD supplemented with LP (20.8 mg/kg BW) and mid-dose PT (41.7 mg/kg BW); and CKDB-322-H, HFD supplemented with LP (20.8 mg/kg BW) and high-dose PT (83.4 mg/kg BW).

Quantitative histological analysis was performed on epididymal adipose tissue sections (Figure 3) to measure the size and number of adipocytes. As shown in Figure 4, quantitative histological analysis revealed that the HFD group exhibited significant adipocyte hypertrophy compared to the ND group, as evidenced by an increase in mean adipocyte size and a decrease in total adipocyte number per field. All treatment groups significantly reduced the average adipocyte size and increased the adipocyte number compared to the HFD group. Notably, CKDB-322-L showed marked increases in the proportion of small adipocytes (<80 μm and 80–100 μm) and the total adipocyte number per field, indicating the greatest reduction in adipocyte hypertrophy and suggesting its superior efficacy in restoring normal adipose tissue architecture.

### 2.8. Effects of CKDB-322 on Gene Expression Related to Adipogenesis and Lipogenesis in Epididymal Adipose Tissue in HFD-Induced Obese Mice

**Figure 5:**
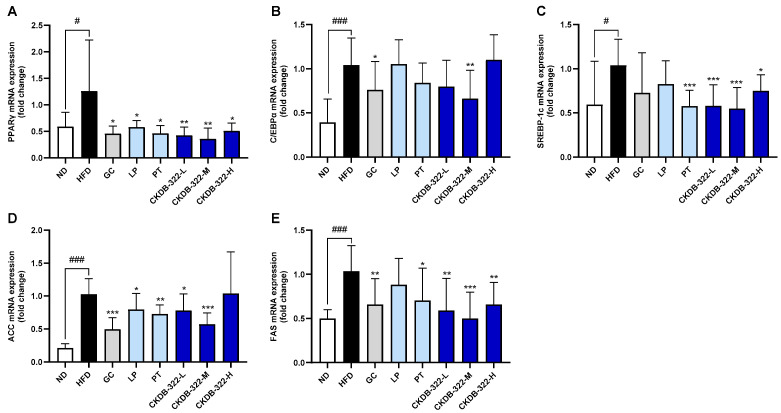
Gene expression analysis of adipogenesis- and lipogenesis-related markers in epididymal adipose tissue of HFD-induced obese mice. The mRNA expression levels of adipogenesis-related genes (**A**,**B**) and lipogenesis-related genes (**C**–**E**) were measured by RT-qPCR using gene-specific primers. Relative expression levels were normalized to β-actin and quantified by densitometric analysis. Data are presented as means ± standard deviations (SD), *n* = 10 mice per group. ^#^ *p* < 0.05, ^###^ *p* < 0.001 indicate significant differences compared to the ND group. * *p* < 0.05, ** *p* < 0.01, and *** *p* < 0.001 indicate significant differences compared to the HFD group. ND, normal-diet group; HFD, 60% high-fat-diet group; GC, HFD supplemented with *Garcinia cambogia* extract (200 mg/kg BW); LP, HFD supplemented with *L. plantarum* Q180 (20.8 mg/kg BW equivalent to 2 × 10^8^ CFU/day); PT, HFD supplemented with *P. tricornutum* (41.7 mg/kg BW); CKDB-322-L, HFD supplemented with LP (20.8 mg/kg BW) and low-dose PT (20.8 mg/kg BW); CKDB-322-M, HFD supplemented with LP (20.8 mg/kg BW) and mid-dose PT (41.7 mg/kg BW); and CKDB-322-H, HFD supplemented with LP (20.8 mg/kg BW) and high-dose PT (83.4 mg/kg BW). PPARγ, peroxisome proliferator-activated receptor gamma; C/EBPα, CCAAT/enhancer-binding protein alpha; SREBP-1c, sterol regulatory element binding protein-1c; ACC, acetyl-CoA carboxylase; and FAS, fatty acid synthase.

As shown in Figure 5, gene expression analysis revealed the significant modulation of genes related to adipogenesis and lipogenesis in the epididymal adipose tissue of HFD-fed mice following treatment. The mRNA expression of PPARγ was significantly increased in the HFD group compared to the ND group (*p* < 0.05), whereas all treatment groups, including GC, LP, PT, and CKDB-322 (L, M, and H), showed significantly reduced expression levels compared to the HFD group (*p* < 0.05 or *p* < 0.01; Figure 5A). Similarly, C/EBPα expression was elevated in the HFD group and significantly downregulated in the LP and CKDB-322-M groups (*p* < 0.05 and *p* < 0.01, respectively), suggesting the suppression of adipogenic signaling (Figure 5B).

For sterol regulatory element-binding protein-1c (SREBP-1c), the HFD group showed increased expression relative to the ND group (*p* < 0.05). PT and all CKDB-322 groups (L, M, and H) exhibited significant reductions in SREBP-1c mRNA levels compared to the HFD group (*p* < 0.001 or *p* < 0.05), indicating a strong inhibitory effect on lipogenic gene transcription (Figure 5C). Regarding lipogenesis-related markers, acetyl-CoA carboxylase (ACC) expression was markedly upregulated in the HFD group (*p* < 0.001) and significantly suppressed by GC, LP, PT, CKDB-322-L, and CKDB-322-M. Notably, GC and CKDB-322-M had the strongest inhibitory effect on ACC expression among all treatment groups (Figure 5D). Finally, fatty acid synthase (FAS) expression was also significantly elevated in the HFD group (*p* < 0.001) and reduced in all treatment groups except the LP one, with CKDB-322-M demonstrating the most substantial downregulation (Figure 5E).

### 2.9. Effects of CKDB-322 on Markers Related to Fatty Acid Oxidation and Energy Metabolism in HFD-Induced Obese Mice

**Figure 6:**
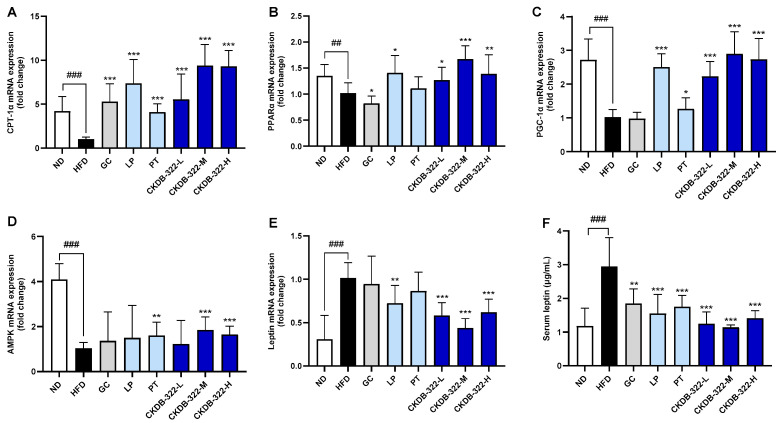
Gene expression analysis of fatty acid oxidation and energy metabolism-related markers in epididymal adipose tissue of HFD-induced obese mice. The mRNA expression levels of fatty acid oxidation-related genes (**A**,**B**) and energy metabolism-related genes (**C**–**E**) in epididymal adipose tissue were measured by RT-qPCR using gene-specific primers. Relative expression levels were normalized to β-actin and quantified by densitometric analysis. Serum leptin levels (**F**) were assessed using appropriate biochemical analysis. Data are presented as means ± standard deviations (SD), *n* = 10 mice per group. ^##^ *p* < 0.01, ^###^ *p* < 0.001 indicates significant differences compared to the ND group. * *p* < 0.05, ** *p* < 0.01, *** *p* < 0.001 indicate significant differences compared to the HFD group. ND, normal-diet group; HFD, 60% high-fat-diet group; GC, HFD supplemented with *Garcinia cambogia* extract (200 mg/kg BW); LP, HFD supplemented with *L. plantarum* Q180 (20.8 mg/kg BW equivalent to 2 × 10^8^ CFU/day); PT, HFD supplemented with *P. tricornutum* (41.7 mg/kg BW); CKDB-322-L, HFD supplemented with LP (20.8 mg/kg BW) and low-dose PT (20.8 mg/kg BW); CKDB-322-M, HFD supplemented with LP (20.8 mg/kg BW) and mid-dose PT (41.7 mg/kg BW); and CKDB-322-H, HFD supplemented with LP (20.8 mg/kg BW) and high-dose PT (83.4 mg/kg BW). CPT-1α, carnitine palmitoyltransferase 1α; PPARα, peroxisome proliferator-activated receptor alpha; PGC-1α, peroxisome proliferator-activated receptor gamma coactivator-1α; and AMPK, 5′-AMP-activated protein kinase.

To evaluate the impact of various treatments on lipid metabolism, mitochondrial function, and energy homeostasis in HFD-induced obese mice, we measured mRNA expression levels of key metabolic genes, which were extracted from epididymal adipose tissues. In addition, serum leptin concentrations were measured across treatment groups. Compared to the ND group, HFD feeding significantly suppressed carnitine palmitoyltransferase 1α (CPT-1α), peroxisome proliferator-activated receptor α (PPARα), PPARγ coactivator-1α (PGC-1α), and 5′-AMP-activated protein kinase (AMPK) mRNA expression levels (Figure 6A–D) while markedly elevating leptin mRNA and serum leptin concentrations (Figure 6E,F). Several treatment groups, including the GC, LP, PT, and CKDB-322 groups, partially reversed these HFD-induced alterations. CPT-1α expression was significantly increased in all treatment groups compared to HFD, with CKDB-322-M and CKDB-322-H showing the greatest enhancement (Figure 6A). Similarly, PPARα expression was restored in the GC, LP, and all CKDB-322 groups, with the most robust increase observed in CKDB-322-M (*p* < 0.001 vs. HFD) (Figure 6B). The groups, including LP or PT, showed improvement in PGC-1α expression, with the CKDB-322-M group in particular reaching a level comparable to the ND group (Figure 6C). The expression of AMPK, which was markedly suppressed in the HFD group, was most significantly restored in the CKDB-322-M and CKDB-322-H groups (*p* < 0.001 vs. HFD) (Figure 6D). All treatments reduced leptin mRNA and serum leptin levels relative to the HFD group. However, CKDB-322-M exhibited one of the most pronounced reductions in both metrics (*p* < 0.001), approaching levels seen in the ND group (Figure 6E,F). Although several interventions showed beneficial effects against HFD-induced metabolic dysfunction, CKDB-322-M consistently demonstrated superior efficacy across all markers—most notably in enhancing CPT-1α, PPARα, and AMPK expression, while effectively reducing leptin levels—highlighting its potential as a therapeutic candidate for metabolic regulation.

### 2.10. Effects of CKDB-322 on Hepatic Lipid Accumulation in HFD-Induced Obese Mice

**Figure 7:**
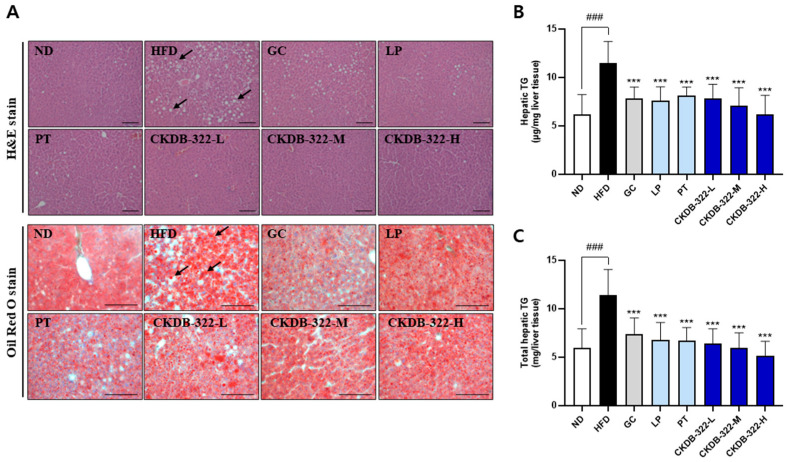
Histological examination of liver and hepatic triglyceride content in HFD-induced obese mice. (**A**) Representative liver sections stained with hematoxylin and eosin (H&E) and Oil Red O (original magnification, 200×; scale bar = 100 µm) are shown for each group. Arrows indicate lipid droplets. (**B**) Hepatic triglyceride levels (µg/mg liver tissue) and (**C**) total hepatic triglyceride content (mg/liver tissue) are quantified for each group. Data are presented as means ± standard deviations (SD), *n* = 10 mice per group. ^###^ *p* < 0.001 indicates significant differences compared to the ND group. *** *p* < 0.001 indicates significant differences compared to the HFD group. ND, normal-diet group; HFD, 60% high-fat-diet group; GC, HFD supplemented with *Garcinia cambogia* extract (200 mg/kg BW); LP, HFD supplemented with *L. plantarum* Q180 (20.8 mg/kg BW equivalent to 2 × 10^8^ CFU/day); PT, HFD supplemented with *P. tricornutum* (41.7 mg/kg BW); CKDB-322-L, HFD supplemented with LP (20.8 mg/kg BW) and low-dose PT (20.8 mg/kg BW); CKDB-322-M, HFD supplemented with LP (20.8 mg/kg BW) and mid-dose PT (41.7 mg/kg BW); and CKDB-322-H, HFD supplemented with LP (20.8 mg/kg BW) and high-dose PT (83.4 mg/kg BW).

As shown in Figure 7A, liver sections from the ND group exhibited a normal hepatic architecture with no evidence of lipid accumulation. In contrast, the HFD group showed substantial fat accumulation and marked hepatic steatosis. Notably, lipid accumulation was markedly reduced in all groups treated with test substances compared to the HFD group, indicating an improvement in hepatic lipid metabolism. Fat accumulation in liver tissue was assessed by Oil Red O staining. The HFD group exhibited numerous red-stained lipid droplets, indicating significant hepatic fat accumulation. In contrast, all test substance-treated groups showed visibly reduced red staining, suggesting the effective suppression of hepatic lipid accumulation. Hepatic TG content was assessed to evaluate lipid accumulation in the liver (Figure 7B,C). The TG concentration per milligram of liver tissue was significantly higher in the HFD group (11.5 ± 2.2 μg/mg) compared to the ND group (6.2 ± 2.0 μg/mg). Similarly, total hepatic TG content was markedly elevated in the HFD group (11.4 ± 2.6 mg/liver) versus the ND group (6.0 ± 1.9 mg/liver). In contrast, all test substance-treated groups exhibited a significant reduction in both hepatic TG concentration and total TG content compared to the HFD group, indicating the attenuation of hepatic lipid accumulation. Notably, the CKDB-322-treated groups showed a dose-dependent reduction in hepatic TG levels.

## 3. Discussion

In this study, we evaluated the anti-obesity effects of CKDB-322, a formulation composed of *L. plantarum* Q180 and *P. tricornutum*, using both in vitro and in vivo models. In 3T3-L1 adipocytes, CKDB-322 significantly inhibited adipogenesis by downregulating key transcription factors (PPARγ and C/EBPα) and enhancing lipolytic activity, as indicated by increased glycerol release. Notably, CKDB-322 exhibited greater efficacy than either component alone in modulating adipocyte metabolism, suggesting a synergistic interaction between the two ingredients. Previous studies have reported that both *L. plantarum* Q180 and *P. tricornutum* individually downregulate adipogenic transcription factors such as PPARγ and C/EBPα in 3T3-L1 adipocytes [17,27]. These findings support the hypothesis that the anti-obesity activity of CKDB-322 may result from synergistic crosstalk between its microbial and microalgal components. Based on these in vitro findings, the effective concentrations of CKDB-322 were used as a reference for dosage selection in subsequent in vivo experiments using HFD-induced obese mice.

CKDB-322 treatment significantly reduced body weight gain, body fat mass, hepatic TG content, and serum levels of glucose and lipids in HFD-induced obese mice models. These effects were more pronounced than those observed with either component alone or the positive control, indicating a potential synergistic interaction between the microalgal and probiotic components. Therefore, the effect of *P. tricornutum* alone was less than that of CKDB-322. The anti-obesity effect of *P. tricornutum* is attributed to its abundance of bioactive hydrophobic substances, such as EPA and fucoxanthin, which are known to modulate lipid metabolism and reduce adiposity [19]. For these reasons, recent research on *P. tricornutum* has focused on optimizing bioactive hydrophobic substance content, enhancing the production of specific fatty acids, and investigating the functions of related genes and proteins. For example, studies on bioactive hydrophobic substance content optimization have shown that adjusting cultivation conditions can significantly increase the production of biomass, lipids, and fucoxanthin [28]. This is an important study that enhances the potential for industrial application through improved productivity. A representative result for increasing the production of unsaturated fatty acids and TG in *P. tricornutum* is that phosphomolybdic acid promotes the production of highly unsaturated fatty acids and TG in *P. tricornutum* [19]. Among the studies on improving lipid content in *P. tricornutum*, some have focused on regulating the expression of enzymes involved in lipid metabolism. A representative example is the study that increased lipid content in *P. tricornutum* by reducing the expression of enoyl-CoA hydratase using the CRISPR interference method [29].

*L. plantarum* Q180 is known for its probiotic activity, including improvements in glucose tolerance, lipid profiles, and gut microbial balance. While improvements in glucose metabolism and intestinal microbial composition have been reported for various *L. plantarum* strains [30,31,32], studies on lipid metabolism have primarily focused on *L. plantarum* Q180. From a clinical perspective, *L. plantarum* Q180 has been shown to influence postprandial lipid metabolism and modulate the gut microbial environment [16]. In particular, *L. plantarum* Q180 administration significantly reduced serum LDL-cholesterol and apolipoprotein levels, as well as postprandial peak concentrations and area-under-the-curve (AUC) values of TG, chylomicron TG, ApoB-48, and ApoB-100. These findings suggest that *L. plantarum* Q180 may exert lipid-lowering effects by improving gut metabolic function. Consistent with this observation, our study demonstrated that *L. plantarum* Q180 effectively suppressed body fat accumulation, exhibiting superior efficacy compared to *P. tricornutum* and comparable outcomes to the positive control, *Garcinia cambogia* extract.

A reduction of up to 55% in hepatic TG levels, along with the attenuation of adipocyte hypertrophy in the CKDB-322 treatment groups, suggests strong potential for alleviating obesity-related hepatic steatosis and systemic inflammation. As shown in Table 3, the TG-lowering effect of CKDB-322 was dose-dependent with respect to the proportion of *P. tricornutum*. These findings indicate that the anti-lipid accumulation effect of the formulation is largely driven by the *P. tricornutum* content. *P. tricornutum* extracts have been reported to exert anti-obesity effects by inhibiting lipogenesis in adipocytes and reducing intracellular lipid accumulation [27]. Given that TGs are the primary form of lipid storage in cells, components of *P. tricornutum* may contribute to TG reduction through multiple physiological mechanisms. One well-documented mechanism is the direct suppression of body fat accumulation by EPA, an omega-3 fatty acid abundantly present in *P. tricornutum* [33]. EPA has been shown to lower blood TG levels by inhibiting TG synthesis and promoting its breakdown [34]. Collectively, these findings suggest that *P. tricornutum* may reduce TG levels by providing bioactive compounds that suppress lipid synthesis and by producing functional lipids like EPA that actively modulate lipid metabolism.

In this experiment, the CKDB-322-L group showed similar effects to the high-dose combination, or in some cases, showed superior effects. The CKDB-322-L group exhibited superior efficacy compared to CKDB-322-M and CKDB-322-H in inhibiting both white adipose tissue accumulation and adipocyte hypertrophy. Regarding metabolic parameters such as blood glucose, TG, and total cholesterol, CKDB-322-L produced effects that were comparable to or marginally better than those of the higher-dose groups.

**Figure 8:**
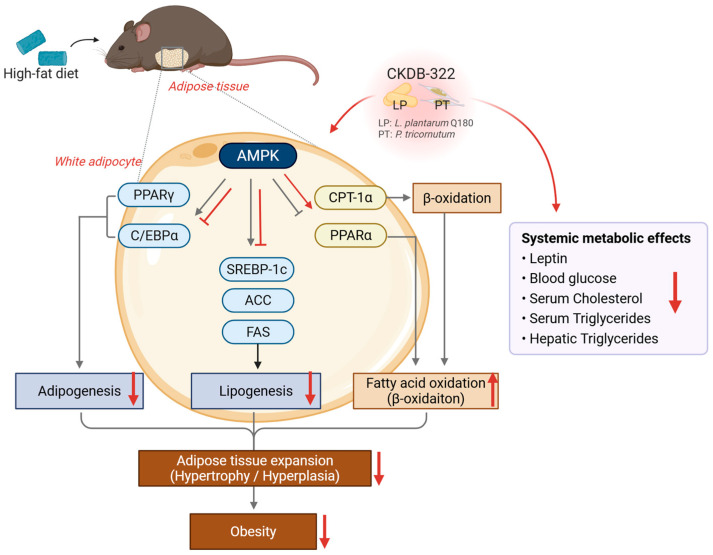
Schematic representation of the molecular mechanisms underlying obesity and the anti-obesity effects of CKDB-322. The high-fat diet induces excessive energy storage in adipose tissue, promoting triglyceride accumulation via AMPK inactivation, which facilitates adipogenesis and lipogenesis while suppressing fatty acid β-oxidation. CKDB-322 supplementation (as indicated by red arrows in the figure) activates AMPK, leading to inhibition of adipogenesis and lipogenesis and stimulation of fatty acid oxidation, thereby suppressing fat accumulation and improving systemic metabolic parameters, including leptin, blood glucose, serum cholesterol, serum triglycerides, and hepatic triglycerides.

At the molecular level, CKDB-322 significantly downregulated the expression of adipogenesis- and lipogenesis-related genes, including PPARγ, C/EBPα, SREBP-1c, ACC, and FAS, in epididymal adipose tissue (Figure 8). Among these, the CKDB-322-M group showed the most pronounced suppression of ACC and FAS, key regulators of lipid synthesis. These in vivo molecular findings are consistent with our in vitro results, where CKDB-322 significantly inhibited adipogenesis in 3T3-L1 adipocytes through the downregulation of PPARγ and C/EBPα. This concordance reinforces the mechanistic rationale for CKDB-322’s anti-obesity effects via the transcriptional regulation of lipid metabolism.

In addition, CKDB-322 enhanced the expression of genes involved in fatty acid oxidation and energy metabolism, such as CPT-1α, PPARα, PGC-1α, and AMPK, particularly in the medium- and higher-dose groups (Figure 8). These transcriptional changes were accompanied by a marked reduction in both mRNA and serum leptin levels, suggesting improved adipose tissue function and systemic energy regulation.

The upregulation of AMPK and PGC-1α implies enhanced mitochondrial biogenesis and fatty acid oxidation, which are key contributors to increased systemic energy expenditure. This observation is consistent with previous findings that *L. plantarum* Q180 activates AMPK/PGC-1α-mediated signaling pathways, thereby improving metabolic efficiency and reducing body fat accumulation [35]. Furthermore, the reduction in leptin expression and circulating levels likely reflects not only decreased adiposity but also improved leptin sensitivity and the restoration of systemic metabolic homeostasis. Given leptin’s central role in energy balance and endocrine regulation [36,37], these findings further support the therapeutic potential of CKDB-322 in obesity management. Importantly, the decrease in leptin levels appears to be a physiological consequence of fat mass reduction rather than an indication of endocrine disruption.

Collectively, these findings suggest that CKDB-322 exerts multi-targeted anti-obesity effects through the coordinated regulation of adipogenesis, lipogenesis, lipolysis, and hepatic lipid metabolism. The consistent efficacy observed in the CKDB-322-M group aligns with the notion that combining probiotic and marine-derived bioactives can produce synergistic metabolic benefits, supporting the potential of CKDB-322 as a novel functional dietary ingredient for obesity management.

Furthermore, although gut microbiota composition and microbial metabolite profiling (e.g., SCFAs) are highly relevant to the mechanism of action of CKDB-322, these analyses were not included in the present in vivo study. This was primarily because the current study focused on evaluating the anti-obesity efficacy and metabolic outcomes of CKDB-322 using physiological, biochemical, and molecular parameters. Comprehensive microbiome and metabolomics analyses are currently under separate investigation as part of our planned follow-up studies aimed at elucidating the underlying mechanisms in greater detail.

The CKDB-322-M formulation used in this animal study, consisting of *L. plantarum* Q180 at 20.8 mg/kg BW and *P. tricornutum* at 41.7 mg/kg BW (a 1:2 weight ratio), was translated into a human-equivalent dose and applied in a subsequent clinical trial. As a result, participants who consumed CKDB-322 exhibited statistically significant reductions in various obesity-related indicators, including body fat mass (g), body fat percentage (%), body weight (g), abdominal fat area (mm^2^), waist circumference (cm), and hip circumference (cm), among other related parameters. These clinical outcomes further support the translational relevance of the present in vivo findings. Importantly, the clinical trial was designed based on the effective mid-dose used in an animal study, targeting endpoints that mirrored the preclinical findings (e.g., body fat reduction and abdominal fat area). This alignment strengthens the biological plausibility and applicability of the preclinical results to human obesity management.

Taken together, these preclinical and clinical findings collectively highlight the potential of CKDB-322 for future industrial development as a functional food or nutraceutical targeting obesity.

## 4. Materials and Methods

### 4.1. Sample Preparation

The probiotic strain *L. plantarum* Q180, originally isolated from the feces of a healthy Korean adult, was provided by Chong Kun Dang Bio Corp. (Ansan, Republic of Korea), and its whole genome sequence is available in the NCBI database under the accession number CP073753. The probiotic powder was obtained by culturing the strain in an optimized medium using a fermenter, followed by concentration and lyophilization. The marine microalga *P. tricornutum* powder used in this study was mass-cultivated in a 50-ton photobioreactor under controlled conditions (temperature 20 ± 2 °C, 2500–3000 lux, pH 7–8) using a modified F/2 + Si medium, and the biomass was harvested by continuous centrifugation, sterilized at 90 °C for 30 min, and freeze-dried [38]. The powder was provided by Microalgae Ask Us Inc. (Gangneung, Republic of Korea). The PT powder was produced by culturing the microalga in an optimized medium using a photobioreactor (PBR), followed by concentration and lyophilization. CKDB-322 was formulated by homogeneously mixing the previously prepared *L. plantarum* Q180 and *P. tricornutum* powders at defined weight ratios, without any further processing. A *Garcinia cambogia* extract (GC), which is well known to have an effect on reducing body fat, was used as a positive control [39]. All test materials, including the positive control, were suspended in distilled water and administered orally.

### 4.2. Characterization of P. tricornutum Using GC-FID

#### 4.2.1. Lipid Extraction from *P. tricornutum*

All lipids were extracted from *P. tricornutum* following a modified Folch method in accordance with the Korean MFDS Food Standards and Specifications [40]. Briefly, 200 mg of homogenized microalgal biomass was placed into a glass tube and mixed with 3 mL of ethanol containing 50 mg of pyrogallol, followed by the addition of 1 mL of internal standard solution (10 mg/mL undecanoic acid methyl ester in chloroform) and 10 mL of 8.3 M hydrochloric acid. The mixture was vortexed and incubated in a water bath at 80 °C for 90 min with periodic vortexing every 10 min. After cooling to room temperature, lipids were extracted twice using a 1:1 (*v*/*v*) mixture of diethyl ether and anhydrous petroleum ether (8 mL per extraction). The ether layers were pooled and evaporated under a gentle stream of nitrogen gas in a 40 °C water bath until viscous.

#### 4.2.2. Derivatization of Fatty Acids into Methyl Esters

The lipid extract was mixed with 3 mL of a 7% boron trifluoride–methanol solution and 1 mL of toluene and then vortexed thoroughly. The mixture was sealed and incubated at 100 °C for 90 min, with intermittent shaking every 30 min to facilitate methylation. After cooling to room temperature, 8 mL of water and 1 mL of n-hexane were added, along with 1 g of anhydrous sodium sulfate. The mixture was vortexed again and centrifuged at 2000 rpm for 20 min. The resulting organic phase was filtered through anhydrous sodium sulfate and transferred to GC vials for analysis. The procedure was performed in accordance with the Korean MFDS Food Standards and Specifications [40]. All reagents used for derivatization were obtained from Sigma-Aldrich (St. Louis, MO, USA).

#### 4.2.3. GC-FID Analysis of Fatty Acid Methyl Esters (FAMEs)

FAMEs were analyzed using a gas chromatograph (GC 8890 Agilent Technologies, Santa Clara, CA, USA) equipped with a flame ionization detector (FID). The procedure was performed in accordance with the Korean MFDS Food Standards and Specifications [40]. Separation was performed on a Supelco SP-2560 column (100 m × 0.25 mm i.d., 0.2 µm film thickness). The oven temperature was initially held at 100 °C for 4 min, then it was ramped up at 3 °C/min to 240 °C and held for 15 min. The injector and detector temperatures were set at 225 °C and 285 °C, respectively. The carrier gas was helium at a flow rate of 0.85 mL/min, and the split ratio was set to 200:1. A 2 µL aliquot of each sample was injected. FAMEs were identified via comparison with authentic standards

### 4.3. In Vitro Experiments

#### 4.3.1. Cell Culture and Adipocyte Differentiation

For this experiment, 3T3-L1 pre-adipocytes were purchased from the American Type Culture Collection (ATCC, Manassas, VA, USA) and cultured at 37 °C in a humidified incubator with 5% CO_2_. Cells were maintained in Dulbecco’s Modified Eagle Medium (DMEM; Gibco, Thermo Fisher Scientific, Waltham, MA, USA) supplemented with 10% (*v*/*v*) bovine calf serum (BCS; HyClone, Logan, UT, USA), 100 U/mL penicillin, and 100 μg/mL streptomycin. When cells reached 60–80% confluence in 6-well plates, they were cultured for an additional 2–4 days in DMEM containing 10% (*v*/*v*) fetal bovine serum (FBS; HyClone, Logan, UT, USA) to induce growth arrest. Adipocyte differentiation was initiated by treating the cells with differentiation medium (DM), consisting of 0.5 mM 3-isobutyl-1-methylxanthine (IBMX; Sigma-Aldrich, St. Louis, MO, USA), 0.25 μM dexamethasone (Sigma-Aldrich), and 10 mg/L insulin (Sigma-Aldrich), for 48 h. The medium was then replaced with DMEM containing 10 mg/L insulin and refreshed every 2 days.

#### 4.3.2. RNA Extraction and RT-qPCR

At the designated time point, 3T3-L1 cells were washed twice with ice-cold phosphate-buffered saline (PBS), harvested, and centrifuged to obtain the cell pellet. Total RNA was isolated using the RNeasy Mini Kit (Qiagen, Hilden, Germany) according to the manufacturer’s instructions. RNA concentration and purity were determined by measuring absorbance at 260 and 280 nm using a microplate spectrophotometer (Epoch™, BioTek Instruments, Winooski, VT, USA), and RNA quality was evaluated based on the A260/A280 ratio.

For cDNA synthesis, 250 ng of total RNA was reverse-transcribed using the PrimeScript™ RT Reagent Kit (TaKaRa, Shiga, Japan). RT-qPCR was conducted using TB Green^®^ Premix Ex Taq™ II (TaKaRa) with the gene-specific primers listed in Table 5. Relative gene expression levels were calculated using the 2^−ΔΔCt^ method. Ct values for each target gene were first normalized to β-actin (ΔCt = Ct_target − Ct_β-actin) and then compared to the appropriate control group (ΔΔCt = ΔCt_treated − ΔCt_control), where the control group was either the DM-only group or the HFD group, depending on the experimental design. Gene expression analysis was performed using RNA samples from 10 biologically independent animals per group. Technical replicates were not conducted in order to prioritize biological variability and statistical robustness. β-actin was selected as the housekeeping gene based on its reported stability in both 3T3-L1 cells and adipose tissue, and its expression in our study showed minimal variation across treatment groups (Ct values ranged from 20.0 to 24.0, with no significant differences, *p* > 0.05). Ct values greater than 35 were considered unreliable and excluded from the analysis.

#### 4.3.3. In Vitro Lipolysis in 3T3-L1 Adipocytes

Differentiated 3T3-L1 adipocytes were treated with the sample for 24 h. After incubation, the supernatants were collected from each well, and glycerol levels were measured using a Glycerol Assay Kit (Sigma-Aldrich) according to the manufacturer’s instructions.

### 4.4. In Vivo Experiments

#### 4.4.1. Animals and Experimental Design

All animal procedures were conducted in accordance with institutional guidelines for the care and use of laboratory animals and were approved by the Institutional Animal Care and Use Committee (IACUC) of Hallym University (Approval No. Hallym 2023-57). Five-week-old male C57BL/6N mice (*n* = 80) were purchased from DooYeol Biotech (Seoul, Republic of Korea) and maintained under specific pathogen-free (SPF) conditions.

Following a one-week acclimatization period under controlled environmental conditions (23 ± 1 °C, 50 ± 5% relative humidity, and 12 h light/dark cycle) with free access to food and water, the mice were randomly assigned to two dietary groups: a normal-diet (ND) group (*n* = 10) and a high-fat-diet (HFD) group (*n* = 70). The ND group received a control diet (D12450B, Research Diets Inc., New Brunswick, NJ, USA) with 10% of the total energy coming from fat, while the HFD group was fed a high-fat diet (D12492, Research Diets Inc.), where 60% of the total energy came from fat, for 8 weeks to induce obesity. The HFD provided 5.1 kcal/g (60% fat, 20% carbohydrate, and 20% protein), whereas the ND provided 3.7 kcal/g (10% fat, 70% carbohydrate, and 20% protein). The HFD-fed mice were then subdivided into the following groups (*n* = 10 per group): HFD control, *Garcinia cambogia* (GC; 200 mg/kg body weight [BW]), LP (20.8 mg/kg BW, equivalent to 2 × 10^8^ CFU/day), PT (41.7 mg/kg BW), CKDB-322-L (LP 20.8 + PT 20.8 mg/kg BW), CKDB-322-M (LP 20.8 + PT 41.7 mg/kg BW), and CKDB-322-H (LP 20.8 + PT 83.4 mg/kg BW). All treatments were administered orally once daily for 8 weeks. Body weight and food intake were recorded weekly.

#### 4.4.2. Biochemical Analysis of Serum

Blood samples were collected from the retro-orbital plexus under isoflurane anesthesia. Serum was separated by centrifugation at 5000 rpm for 10 min at 4 °C and analyzed for glucose, triglycerides (TGs), total cholesterol (TC), high-density lipoprotein cholesterol (HDL-C), low-density lipoprotein cholesterol (LDL-C), alanine aminotransferase (ALT), and aspartate aminotransferase (AST) using an automated biochemical analyzer (KoneLab 20 XT, Thermo Fisher Scientific, Waltham, MA, USA).

#### 4.4.3. Body Composition Analysis by Dual-Energy X-Ray Absorptiometry (DEXA)

At the end of the experimental period, body composition, including fat mass, was measured using a DEXA system (PIXImus™, GE Lunar, Madison, WI, USA).

#### 4.4.4. Tissue Collection and Weight Measurement

White adipose tissues (WATs), including epididymal, visceral, retroperitoneal, and inguinal fat pads, as well as the liver, were carefully excised, weighed, and immediately stored at −70 °C for further analysis.

#### 4.4.5. Histological Analysis

Epididymal adipose tissue and liver samples were fixed in 4% paraformaldehyde and embedded in either paraffin or optimal cutting temperature (OCT) compound. Tissue sections (5–10 μm) were stained with hematoxylin and eosin (H&E) or Oil Red O. Histological features were observed under a light microscope (Carl Zeiss, Oberkochen, Germany).

#### 4.4.6. Evaluation of Adipocyte Size and Number in Epididymal Adipose Tissue

Adipocyte size and number were quantified from H&E-stained images of epididymal adipose tissue using an AxioVision Imaging Analysis System (Carl Zeiss, Oberkochen, Germany).

#### 4.4.7. RNA Extraction and RT-qPCR

Total RNA was extracted from epididymal adipose tissue using the same procedures described for the in vitro experiment. Reverse transcription and RT-qPCR were performed as previously described in Section 4.3.2. The gene-specific primers used in this study are listed in Table 5.

#### 4.4.8. Hepatic TG Content

Liver tissues were homogenized, and lipids were extracted using a modified Folch method. Hepatic TG levels were measured using a commercial enzymatic assay kit (Asan Pharmaceutical, Seoul, Republic of Korea) and expressed as both μg/mg tissue and mg/whole liver.

### 4.5. Statistical Analysis

All statistical analyses were performed using GraphPad Prism version 10.5.0 (GraphPad Software, San Diego, CA, USA). Data are presented as the means ± standard deviations (SD), with *n* = 3 (in vitro experiments) or 10 (animal experiments) per group. Statistical significance was evaluated by one-way analysis of variance (ANOVA) followed by Tukey’s multiple comparison test. ^#^ *p* < 0.05, ^##^ *p* < 0.01, and ^###^ *p* < 0.001 indicate significant differences compared to the undifferentiated control (pre-adipocytes) or the ND group. * *p* < 0.05, ** *p* < 0.01, and *** *p* < 0.001 indicate significant differences compared to the DM-only group (differentiated adipocytes) or the HFD group.

## 5. Conclusions

CKDB-322, a formulation composed of *L. plantarum* Q180 and *P. tricornutum*, significantly improved obesity-related parameters in HFD-induced obese mice, including reductions in body weight gain, adiposity, hepatic lipid accumulation, and serum metabolic markers. Compared to either component alone or the positive control, the mixture demonstrated superior efficacy, suggesting a synergistic interaction between its constituents. These findings indicate that a mixture of probiotics and marine microalgae may represent a promising natural intervention for obesity management. CKDB-322 holds potential for development as a functional ingredient in health supplements or metabolism-targeted functional foods. However, this study has certain limitations: While gut microbiota composition and microbial metabolite profiling are supposed to be highly relevant to the mechanism of action of CKDB-322, these analyses were not included in the present in vivo study. In addition, mechanistic pathways such as gene expression, microbiota composition, and inflammatory markers were also not assessed. Further studies are warranted to confirm these effects in clinical settings and to elucidate the molecular basis underlying the observed synergy. Mechanistic pathways such as gene expression, microbiota composition, and inflammatory markers were not assessed. Further studies are warranted to confirm these effects in clinical settings and to elucidate the molecular basis underlying the observed synergy.

## Figures and Tables

**Table 1:** Fatty acid composition of *P. tricornutum* as determined by GC-FID (mg/100 g DW).

Peak No.	Name		Contents (mg/100 g)
1	Butyric acid	C4:0	0.00
2	Caproic acid	C6:0	0.00
3	Caprylic acid	C8:0	0.00
4	Capric acid	C10:0	3.40 ± 0.17
5	Undecanoic acid	C11:0	0.00
6	Lauric acid	C12:0	29.23 ± 50.63
7	Tridecanoic acid	C13:0	0.00
8	Myristic acid	C14:0	936.07 ± 26.95
9	Myristoleic acid	C14:1	51.70 ± 20.74
10	Pentadecylic acid	C15:0	31.30 ± 0.82
11	Cis-Pentadecenoic acid	C15:1	0.00
12	Palmitic acid	C16:0	1290.00 ± 41.62
13	Palmitoleic acid	C16:1n-7	2725.07 ± 76.21
14	Heptadecanoic acid	C17:0	12.47 ± 0.40
15	Cis-Heptadecenoic acid	C17:1	20.23 ± 0.60
16	Stearic acid	C18:0	17.93 ± 0.47
17	Trans-Oleic acid	C18:1-trans	12.70 ± 0.72
18	Oleic acid	C18:1n-9	1219.23 ± 49.16
19	Trans-Linoleic acid	C18:2-trans	82.47 ± 1.25
20	Linoleic acid	C18:2n-6	572.73 ± 17.67
21	Arachidic acid	C20:0	4.53 ± 0.06
22	Gamma-linolenic acid (GLA)	C18:3n-6	73.77 ± 1.96
23	Cis-11-eicosenoic acid	C20:1n-9	20.50 ± 0.56
24	Alpha-linolenic acid (ALA)	C18:3n-3	28.67 ± 0.50
25	Heneicosanoic acid	C21:0	0.00
26	Eicosadienoic acid	C20:2n-6	71.57 ± 1.92
27	Behenic acid	C22:0	36.20 ± 1.21
28	Cis-11,14,17-Eicosatrienoic acid	C20:3n-3	20.80 ± 6.17
29	Cis-13-docosenoic acid	C22:1n-9	72.33 ± 8.30
30	Nonadecanoic acid	C19:0	0.00
31	Tricosanoic acid	C23:0	0.00
32	Arachidonic acid (AA)	C20:4n-6	372.53 ± 49.79
33	Docosadienoic acid	C22:2n-6	35.67 ± 0.80
34	Lignoceric acid	C24:0	221.83 ± 8.27
35	Eicosapentaenoic acid (EPA)	C20:5n-3	3194.23 ± 81.27
36	Nervonic acid	C24:1n-9	54.27 ± 2.59
37	Docosahexaenoic acid (DHA)	C22:6n-3	300.37 ± 5.77
	Total		11,511.67 ± 288.35

**Table 2:** Effects of CKDB-322 on body weight, weight gain, food intake, and FER in HFD-induced obese mice.

Parameters	ND	HFD-Induced Obese Mice						
		HFD	GC	LP	PT	CKDB-322-L	CKDB-322-M	CKDB-322-H
Initial body weight (g)	22.2 ± 1.2	22.6 ± 0.6	22.4 ± 1.3	22.4 ± 0.8	22.8 ± 1.2	22.5 ± 1.1	22.0 ± 0.9	22.3 ± 0.8
Final body weight (g)	29.3 ± 1.4	39.0 ± 3.0 ^###^	34.5 ± 2.3 **	34.2 ± 1.8 ***	36.0 ± 1.7 *	33.5 ± 1.6 ***	33.9 ± 2.1 ***	33.0 ± 2.2 ***
Weight gain ^1^ (g)	7.1 ± 1.9	16.4 ± 3.2 ^###^	12.1 ± 2.7 **	11.8 ± 1.7 ***	13.2 ± 2.2 *	11.0 ± 1.4 ***	11.9 ± 1.9 ***	10.7 ± 2.2 ***
Food intake (g/day)	2.8 ± 0.2	2.2 ± 0.0 ^###^	2.2 ± 0.1	2.1 ± 0.0 ***	2.1 ± 0.0 ***	2.1 ± 0.1 ***	2.1 ± 0.1 ***	2.1 ± 0.1 ***
FER ^2^	0.047 ± 0.0	0.133 ± 0.0 ^###^	0.100 ± 0.0 **	0.100 ± 0.0 **	0.112 ± 0.0	0.094 ± 0.0 ***	0.102 ± 0.0 **	0.092 ± 0.0 ***

^1^ Weight gain was calculated as the difference between final and initial body weight. ^2^ Feed efficiency ratio (FER) was calculated as FER = (weight gain (g)/cumulative food intake (g)) × 100. Values are expressed as means ± standard deviations (SD), with *n* = 10 mice per group. ^###^ *p* < 0.001 indicates significant differences compared to the ND group. * *p* < 0.05, ** *p* < 0.01, and *** *p* < 0.001 indicate significant differences compared to the HFD group. ND, normal-diet group; HFD, 60% high-fat-diet group; GC, HFD supplemented with *Garcinia cambogia* extract (200 mg/kg BW); LP, HFD supplemented with *L. plantarum* Q180 (20.8 mg/kg BW equivalent to 2 × 10^8^ CFU/day); PT, HFD supplemented with *P. tricornutum* (41.7 mg/kg BW); CKDB-322-L, HFD supplemented with LP (20.8 mg/kg BW) and low-dose PT (20.8 mg/kg BW); CKDB-322-M, HFD supplemented with LP (20.8 mg/kg BW) and mid-dose PT (41.7 mg/kg BW); and CKDB-322-H, HFD supplemented with LP (20.8 mg/kg BW) and high-dose PT (83.4 mg/kg BW).

**Table 3:** Effects of CKDB-322 on serum biochemical parameters in HFD-induced obese mice.

Parameters	ND	HFD-Induced Obese Mice						
		HFD	GC	LP	PT	CKDB-322-L	CKDB-322-M	CKDB-322-H
Glucose (mg/dL)	168.5 ± 36.7	221.9 ± 23.9 ^##^	168.1 ± 36.8 **	198.8 ± 30.1	193.3 ± 31.2 *	177.0 ± 36.6 **	172.3 ± 17.9 ***	256.8 ± 45.8
TG (mg/dL)	47.1 ± 8.9	61.1 ± 6.4 ^###^	67.3 ± 16.1	61.9 ± 23.3	62.6 ± 5.7	49.8 ± 5.4 ***	42.6 ± 9.2 ***	44.5 ± 1.1 ***
Total cholesterol (mg/dL)	106.2 ± 12.4	149.0 ± 9.0 ^###^	120.9 ± 25.5 **	119.2 ± 12.1 ***	142.5 ± 16.4	119.1 ± 21.3 ***	117.3 ± 12.5 ***	129.1 ± 15.8 **
LDL-cholesterol (mg/dL)	10.8 ± 2.7	18.0 ± 3.8 ^###^	15.5 ± 3.6	14.7 ± 4.0	18.3 ± 3.8	13.5 ± 3.3 *	14.4 ± 3.8 *	16.4 ± 3.2
HDL-cholesterol (mg/dL)	97.0 ± 7.7	134.4 ± 10.0 ^###^	106.9 ± 15.4 ***	104.0 ± 14.2 ***	125.0 ± 18.4	116.3 ± 10.9 **	115.3 ± 10.4 ***	123.4 ± 17.5
AST (U/L)	110.9 ± 21.5	121.5 ± 32.8	118.1 ± 24.4	111.9 ± 28.3	114.2 ± 24.5	118.6 ± 32.6	104.1 ± 19.5	113.0 ± 27.5
ALT (U/L)	61.0 ± 22.0	86.3 ± 33.0	76.0 ± 37.4	65.6 ± 24.9	76.1 ± 19.7	64.3 ± 29.0	70.0 ± 20.5	82.1 ± 59.9

Values are expressed as means ± standard deviations (SD), with *n* = 10 mice per group. ^##^ *p* <0.01, ^###^ *p* < 0.001 indicate significant differences compared to the ND group. * *p* < 0.05, ** *p* < 0.01, and *** *p* < 0.001 indicate significant differences compared to the HFD group. ND, normal-diet group; HFD, 60% high-fat-diet group; GC, HFD supplemented with Garcinia cambogia extract (200 mg/kg BW); LP, HFD supplemented with *L. plantarum* Q180 (20.8 mg/kg BW equivalent to 2 × 10^8^ CFU/day); PT, HFD supplemented with *P. tricornutum* (41.7 mg/kg BW); CKDB-322-L, HFD supplemented with LP (20.8 mg/kg BW) and low-dose PT (20.8 mg/kg BW); CKDB-322-M, HFD supplemented with LP (20.8 mg/kg BW) and mid-dose PT (41.7 mg/kg BW); CKDB-322-H, HFD supplemented with LP (20.8 mg/kg BW) and high-dose PT (83.4 mg/kg BW). LDL-cholesterol, low-density lipoprotein cholesterol; HDL-cholesterol, high-density lipoprotein cholesterol; AST, aspartate aminotransferase; and ALT, alanine aminotransferase.

**Table 4:** Effects of CKDB-322 on body fat and organ weights in HFD-induced obese mice.

Parameters	ND	HFD-Induced Obese Mice						
		HFD	GC	LP	PT	CKDB-322-L	CKDB-322-M	CKDB-322-H
Fat in tissue ^1^ (%)	25.2 ± 3.6	40.5 ± 2.0 ^###^	33.9 ± 5.1 **	34.9 ± 2.7 ***	37.1 ± 4.2 *	32.8 ± 4.6 ***	35.2 ± 3.2 ***	34.0 ± 4.0 ***
White adipose tissue (g)								
Total weight	1.53 ± 0.44	4.75 ± 0.91 ^###^	3.15 ± 0.88 ***	3.28 ± 0.73 ***	4.00 ± 0.76	2.95 ± 0.67 ***	3.29 ± 0.50 ***	3.24 ± 0.65 ***
Epididymal fat	0.78 ± 0.23	2.44 ± 0.41 ^###^	1.63 ± 0.48 ***	1.67 ± 0.46 ***	2.04 ± 0.44	1.50 ± 0.39 ***	1.71 ± 0.29 ***	1.61 ± 0.33 ***
Retroperitoneal fat	0.32 ± 0.10	0.94 ± 0.12 ^###^	0.69 ± 0.18 **	0.75 ± 0.15 **	0.91 ± 0.17	0.62 ± 0.10 ***	0.71 ± 0.13 ***	0.71 ± 0.14 ***
Mesenteric fat	0.25 ± 0.07	0.77 ± 0.33 ^###^	0.42 ± 0.16 **	0.41 ± 0.11 **	0.52 ± 0.12 *	0.41 ± 0.12 **	0.42 ± 0.10 **	0.47 ± 0.16 *
Inguinal fat	0.18 ± 0.08	0.59 ± 0.12 ^###^	0.41 ± 0.12 **	0.45 ± 0.09 *	0.54 ± 0.12	0.42 ± 0.14 **	0.45 ± 0.07 **	0.45 ± 0.12 *
Liver (g)	0.95 ± 0.04	1.06 ± 0.12 ^#^	0.90 ± 0.06 **	0.88 ± 0.10 **	0.86 ± 0.06 ***	0.80 ± 0.07 ***	0.81 ± 0.05 ***	0.84 ± 0.05 ***

^1^ Measured using dual-energy X-ray absorptiometry (DEXA). Values are expressed as means ± standard deviations (SD), with *n* = 10 mice per group. ^#^ *p* < 0.05, ^###^ *p* < 0.001 indicate significant differences compared to the ND group. * *p* < 0.05, ** *p* < 0.01, and *** *p* < 0.001 indicate significant differences compared to the HFD group. ND, normal-diet group; HFD, 60% high-fat-diet group; GC, HFD supplemented with *Garcinia cambogia* extract (200 mg/kg BW); LP, HFD supplemented with *L. plantarum* Q180 (20.8 mg/kg BW equivalent to 2 × 10^8^ CFU/day); PT, HFD supplemented with *P. tricornutum* (41.7 mg/kg BW); CKDB-322-L, HFD supplemented with LP (20.8 mg/kg BW) and low-dose PT (20.8 mg/kg BW); CKDB-322-M, HFD supplemented with LP (20.8 mg/kg BW) and mid-dose PT (41.7 mg/kg BW); and CKDB-322-H, HFD supplemented with LP (20.8 mg/kg BW) and high-dose PT (83.4 mg/kg BW).

**Table 5:** Primer sequences used for the real-time PCR quantification of mRNA expression.

Gene	Primer Sequence	
PPARγ	F:	5′-GCCCACCAACTTCGGAATC-3′
	R:	5′-TGCGAGTGGTCTTCCATCAC-3′
C/EBPα	F:	5′-GAGCTGAGTGAGGCTCTCATTCT-3′
	R:	5′-TGGGAGGCAGACGAAAAAAC-3′
SREBP-1c	F:	5′-CCAGAGGGTGAGCCTGACAA-3′
	R:	5′-AGCCTCTGCAATTTCCAGATCT-3′
ACC	F:	5′-CGAGTCCTCTCCTCAGCTCC-3′
	R:	5′-ATCGGGAGTGCTGGTTTAGC-3′
FAS	F:	5′-CAAGTGTCCACCAACAAGCG-3′
	R:	5′-GGAGCGCAGGATAGACTCAC-3′
CPT-1α	F:	5′-GACTCCGCTCGCTCATTCC-3′
	R:	5′-ACGCCACTCACGATGTTCTT-3′
PPARα	F:	5′-CCGAACATTGGTGTTCGCAG-3′
	R:	5′-AGATACGCCCAAATGCACCA-3′
PGC-1α	F:	5′-TCTCAGTAAGGGGCTGGTTG-3′
	R:	5′-TTCCGATTGGTCGCTACACC-3′
AMPK	F:	5′-AGCCCTTCCTTCTCTTGCTC-3′
	R:	5′-AGGATGCCTGAAAAGCTTGA-3′
Leptin	F:	5′-GAGACCCCTGTGTCGGTTC-3′
	R:	5′-CTGCGTGTGTGAAATGTCATTG-3′
β-actin	F:	5′-CATTGCTGACAGGATGCAGAAGG-3′
	R:	5′-TGCTGGAAGGTGGACAGTGAGG-3′

PPARγ, peroxisome proliferator-activated receptor gamma; C/EBPα, CCAAT/enhancer-binding protein alpha; SREBP-1c, sterol regulatory element binding protein-1c; ACC, Acetyl-CoA carboxylase; FAS, fatty acid synthase; CPT-1α, carnitine palmitoyltransferase 1α; PPARα, peroxisome proliferator-activated receptor alpha; PGC-1α, peroxisome proliferator-activated receptor gamma coactivator-1α; and AMPK, 5′-AMP-activated protein kinase.

## Data Availability

The data presented in this study are available from the corresponding author upon reasonable request. We would like to clarify that the research data underlying this study cannot be publicly shared due to restrictions specified in the formal collaboration agreement between the two participating institutions. The agreement includes clauses regarding data ownership and confidentiality, which prohibit public dissemination without prior institutional approval. However, the data can be made available by the corresponding author upon reasonable request and with appropriate institutional authorization.

## Supplementary Materials

The following supporting information can be downloaded at https://www.mdpi.com/article/10.3390/ijms26167991/s1.
