# Supplementary material for: Synergistic Anti-Obesity Effects of Lactiplantibacillus plantarum Q180 and Phaeodactylum tricornutum (CKDB-322) in High-Fat-Diet-Induced Obese Mice
Source: Int J Mol Sci. 2025 Aug 19;26(16):7991. doi: 10.3390/ijms26167991 (PMC12386854; doi:10.3390/ijms26167991)
Supplement: Supplementary file 1 [file ijms-26-07991-s001.zip › ijms-3771435-supplementary.pdf]

International Journal of Molecular Sciences

MDPI

Supplementary Table S1. Raw data corresponding to Figure 1. mRNA expression levels of adipogenic transcription factors PPARγ and C/EBPα measured by qRT-PCR and expressed as relative values using the 2^-ΔΔCT method. Values are presented as mean ± SE (n = 3).

Group	PPARγ	C/EBPα
undifferentiated control(pre-adipocyte)	0.305 ± 0.0184	0.4874 ± 0.0846
DM-only group(differentiated adipocyte)	1.0007 ± 0.0457	1.0007 ± 0.0448
L. plantarum Q180 0.29 mg/mL (1 x 10^8 CFU/mL equivalent)	0.9292 ± 0.0134	1.0469 ± 0.0323
L. plantarum Q180 0.58 mg/mL (2 x 10^8 CFU/mL equivalent)	0.8683 ± 0.0885	1.0642 ± 0.1501
L. plantarum Q180 1.16 mg/mL (4 x 10^8 CFU/mL equivalent)	0.7662 ± 0.1420	1.0741 ± 0.1776
P. tricornutum 0.58 mg/mL	0.7018 ± 0.0142	1.1658 ± 0.3126
P. tricornutum 1.16 mg/mL	0.6855 ± 0.0355	0.8646 ± 0.0373
P. tricornutum 2.32 mg/mL	0.7393 ± 0.0037	0.8841 ± 0.0978
L. plantarum Q180 0.58 mg/mL + *P. tricornutum* 0.58 mg/mL (1:1 vol)	0.3741 ± 0.0065	0.7046 ± 0.0237
L. plantarum Q180 0.58 mg/mL + *P. tricornutum* 1.16 mg/mL (1:2 vol)	0.3032 ± 0.0117	0.3808 ± 0.0279
L. plantarum Q180 0.58 mg/mL + *P. tricornutum* 2.32 mg/mL (1:4 vol)	0.4024 ± 0.0105	0.5058 ± 0.0194

International Journal of Molecular Sciences

MDPI

Supplementary Table S2. Raw data corresponding to Figure 2. Glycerol release (μg/mL) results. Values are presented as mean ± SE (n = 3).

Group	Glyceorl release (μg/mL)
undifferentiated control(pre-adipocyte)	3.217 ± 0.058
DM-only group(differentiated adipocyte)	5.647 ± 0.038
L. plantarum Q180 0.075 mg/mL (2.5 x 10^7 CFU/mL equivalent)	5.554 ± 0.014
L. plantarum Q180 0.145 mg/mL (5 x 10^7 CFU/mL equivalent)	6.210 ± 0.014
L. plantarum Q180 0.29 mg/mL (1 x 10^8 CFU/mL equivalent)	6.542 ± 0.141
L. plantarum Q180 0.58 mg/mL (2 x 10^8 CFU/mL equivalent)	7.129 ± 0.098
L. plantarum Q180 1.16 mg/mL (4 x 10^8 CFU/mL equivalent)	5.502 ± 0.093
P. tricornutum 0.145 mg/mL	7.334 ± 0.137
P. tricornutum 0.29 mg/mL	7.708 ± 0.190
P. tricornutum 0.58 mg/mL	7.858 ± 0.093
P. tricornutum 1.16 mg/mL	8.721 ± 0.023
P. tricornutum 2.32 mg/mL	7.337 ± 0.290
L. plantarum Q180 0.58 mg/mL + *P. tricornutum* 0.58 mg/mL (1:1 vol)	13.671 ± 0.528
L. plantarum Q180 0.58 mg/mL + *P. tricornutum* 1.16 mg/mL (1:2 vol)	16.035 ± 0.042
L. plantarum Q180 0.58 mg/mL + *P. tricornutum* 2.32 mg/mL (1:4 vol)	16.019 ± 0.127

 https://doi.org/10.3390/ijms26167991
